# Effects of contact stress on patellarfemoral joint and quadriceps force in fixed and mobile-bearing medial unicompartmental knee arthroplasty

**DOI:** 10.1186/s13018-020-02047-0

**Published:** 2020-11-10

**Authors:** Hyuck Min Kwon, Jin-Ah Lee, Yong-Gon Koh, Kwan Kyu Park, Kyoung-Tak Kang

**Affiliations:** 1 Department of Orthopedic Surgery, Yonsei University College of Medicine, 50-1 Yonsei-ro, Seodaemun-gu, Seoul, 03722 Republic of Korea; 2 Department of Mechanical Engineering, Yonsei University, 50 Yonsei-ro, Seodaemun-gu, Seoul, 03722 Republic of Korea; 3 Joint Reconstruction Center, Department of Orthopaedic Surgery, Yonsei Sarang Hospital, 10 Hyoryeong-ro, Seocho-gu, Seoul, 06698 Republic of Korea

**Keywords:** Finite-element method, Unicompartmental knee arthroplasty, Patellofemoral joint

## Abstract

**Background:**

Unicompartmental knee arthroplasty (UKA) is an effective treatment for end-stage, symptomatic unicompartmental osteoarthritis of the knee joint. However, patellofemoral joint degeneration is a contraindication to medial UKA. Therefore, the objective of this study was to evaluate the biomechanical effect of medial UKA using fixed-bearing (FB) and mobile-bearing (MB) design prostheses on the patellofemoral joint.

**Methods:**

A three-dimensional finite-element model of a normal knee joint was developed using medical image data. We performed statistical analysis for each model. The differences in contact stress on the patellofemoral joint and the quadriceps force between the FB and MB designs were evaluated under a deep-knee-bend condition.

**Results:**

At an early flexion angle, the results of contact stress showed no significant difference between the FB and MB medial UKA models compared with the intact model. However, at high flexion angles, we observed a significant increase in contact stress with the FB models compared with the intact model. On the contrary, in the case of the MB models, we found no statistically significant increment compared with the intact model. A larger quadriceps force was needed to produce an identical flexion angle for both the FB and MB UKA designs than for the intact model. At high flexion angles, a significant increase quadriceps force whit the FB model compared with the intact model.

**Conclusions:**

Our results indicate that with medial UKA, the contact stress increased and greater quadriceps force was applied to the patellofemoral joint. However, performing UKA on a patellofemoral joint with osteoarthritis should not be difficult, unless anterior knee pain is present, because the increase in contact stress is negligible.

## Background

Unicompartmental knee arthroplasty (UKA) is a surgical treatment alternative to total knee arthroplasty for isolated medial compartmental arthritis of the knee joint. The benefits of UKA include fewer complications, faster recovery, improved functional outcomes, and cost-effectiveness [1–4]. Therefore, medial UKA had been increasingly used for the treatment of medial compartmental osteoarthritis (OA) over the past two decades [5]. Historically, patellofemoral (PF) joint degeneration, and more specifically, advanced lateral PF joint facet degeneration, along with anterior knee pain, has been considered as an exclusion criterion for medial UKA [6, 7]. However, PF joint degeneration has been reported recently to have no influence on the clinical outcomes after UKA [8, 9]. In addition, whether preexisting PF joint degeneration is a contraindication to UKA is controversial. Thein et al. recently performed a study to determine the effect of medial fixed-bearing (FB) UKA on postoperative PF joint congruence and analyzed the effect of preoperative PF joint degeneration on the clinical outcome [10]. No correlation was observed between preoperative PF joint congruence or degeneration severity and the Western Ontario and McMaster Universities Osteoarthritis Index (WOMAC) scores at 2-year follow-up [10]. Preoperative PF joint congruence and degenerative changes do not affect the clinical outcomes after UKA [10]. However, multiple studies that used the Oxford knee system indicated that neither preoperative anterior knee pain nor moderate radiological PF osteoarthritic changes affected the long-term clinical outcomes and survivorship of patients after mobile-bearing (MB) UKA [9, 11, 12]. One study suggested that MB UKA provides better restoration of normal knee kinematics, which theoretically translates to better patellar tracking and long-term outcomes [13]. Although several studies have revealed no significant differences in clinical outcomes and complication rates between the FB and MB UKA designs, the mode of failure often differs [14]. In addition, research on the biomechanical effect of medial UKA on the PF joint is lacking. The biomechanical effect on the PF joint can be investigated by performing a finite-element (FE) analysis to evaluate the contact stress and quadriceps force after medial UKA [15]. Accurate *in silico* evaluations of knee joint replacements are useful for clinical assessment [15].

Therefore, the objective of this study was to evaluate the biomechanical effects of medial UKA using FB and MB design prostheses on the PF joint. The differences in contact stress on the PF joint and quadriceps force between the FB and MB designs were evaluated under a deep-knee-bend condition. We hypothesized that applying medial UKA would not be difficult even with OA of the PF joint (unless accompanied by anterior knee pain) because the differences in biomechanical effect on the PF joint are negligible between the UKA and normal knee joint models.

## Methods

### Normal knee joint model

In this study, an existing three-dimensional non-linear FE model of the knee joint based on data from four male subjects (subject 1: age, 36 years; height, 178 cm; mass, 75 kg; subject 2: age, 34 years; height, 173 cm; mass, 83 kg; subject 3: age, 32 years; height, 182 cm; mass, 79 kg; subject 4: age, 34 years; height, 173 cm; mass, 71 kg) and one female subject (subject 5: age, 26 years; height, 163 cm; mass, 65 kg) was used. The FE model was developed using computed tomography and magnetic resonance imaging data with a slice thikness of 0.1 mm and 0.4 mm slice, respectively [16, 17] and included the bony structures of the knee joint and the soft tissues of the PF and tibiofemoral (TF) joint anatomies. The articular cartilage and menisci were defined as isotropic linearly elastic materials and transversely isotropic and linearly elastic materials, respectively [18]. The material properties of the articular cartilage and menisci are presented in Table 1.

**Table 1:** Material properties of the articular cartilage and menisci

Cartilage	Linearly elastic, isotropic	*E* = 15 MPa *v* = 0.475
Menisci	Linearly elastic, transversely isotropic	*E*_*θ*_ = 150 MPa, *E*_*r*_ = *E*_*z*_ = 20 MPa *v*_*rz*_ = 0.2, *v*_*rθ*_ = *v*_*zθ*_ = 0.3, *G*_*rθ*_ = *G*_*zθ*_ = 57.7 MPa

All the ligaments were modeled with nonlinear and tension-only spring elements [19, 20]. Mesh convergence tests were performed to complete the simulation. Convergence was obtained if the relative change between two adjacent meshes was < 5%. The average element sizes were 0.8 mm for the cartilage and menisci, respectively. The details of the meshes used in the FE model were described in Table 2. The interfaces between the cartilage and bones were modeled to be fully bonded. Contact was applied between the femoral cartilage and meniscus, meniscus and tibial cartilage, and femoral cartilage and tibial cartilage for both the medial and lateral sides [16].

**Table 2:** Details of meshes used in the FE model

Set	Nomal knee	FB UKA	MB UKA
Femur bone (Quad)	18,817	17,948	17,899
Tibia bone (Quad)	13,286	12,303	12,289
Fibula bone (Quad)	5456	5456	5456
Patella bone (Hexa)	1411	1411	1411
Femur cartilage (Hexa)	9629	4586	4586
Meniscus (Hexa)	2978	1475	1475
Femoral componennt (Hexa)	–	7335	7523
PE insert (Hexa)	–	2872	3125
Tibia tray (Hexa)	–	2421	3094
Total	51,577	55,807	56,858

### Medial UKA model

**Fig. 1:**
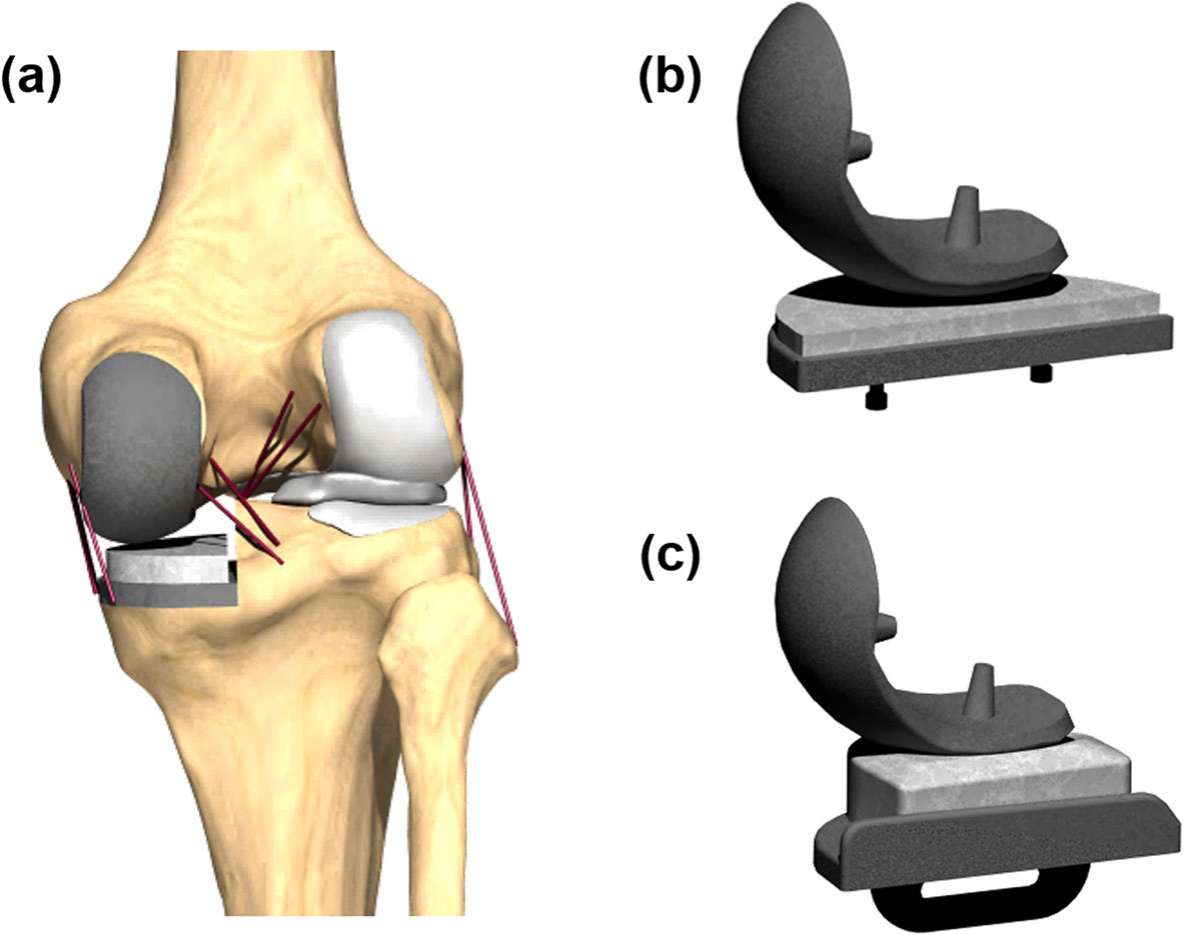
FE model used in this study for **a** UKA model, **b** FB UKA componenet design, and **c** MB UKA componenet design

A FB UKA prosthesis (Zimmer, Inc., Warsaw, IN, USA) and a MB UKA prosthesis of the Oxford knee system (Biomet, Warsaw, IN) were virtually implanted in the medial compartment of the normal knee joint model. (Fig. 1) The bone models were imported and appropriately positioned, trimmed, and meshed with rigid elements according to the surgical techniques [16]. The tibial component was defined as a square (0°) inclination in the coronal plane, with a 5° posterior slope. The rotating axis was defined as a line parallel to the lateral edge of the tibial component passing through the center of the femoral component peg. A femoral component distal cut perpendicular to the mechanical axis of the femur and parallel to the tibial cut was reproduced. The height of the PE insert was identical to the anatomy in a sagittal plane aligned with the mechanical axis of the tibia and positioned at the medial edge of the tibia. The materials used for the femoral component, tibial insert, tibial baseplate, and bone cement were cobalt chromium molybdenum alloy, ultrahigh-molecular-weight polyethylene, titanium alloy, and polymethyl methacrylate, respectively (Table 3) [17, 21, 22].

**Table 3:** Material properties of implant

	Young’s modulus (MPa)	Poisson’s ratio
CoCr alloy	220,000	0.30
UHMWPE	685	0.47
Ti6AI4V alloy	110,000	0.30
PMMA	1,940	0.4

**Fig. 2:**
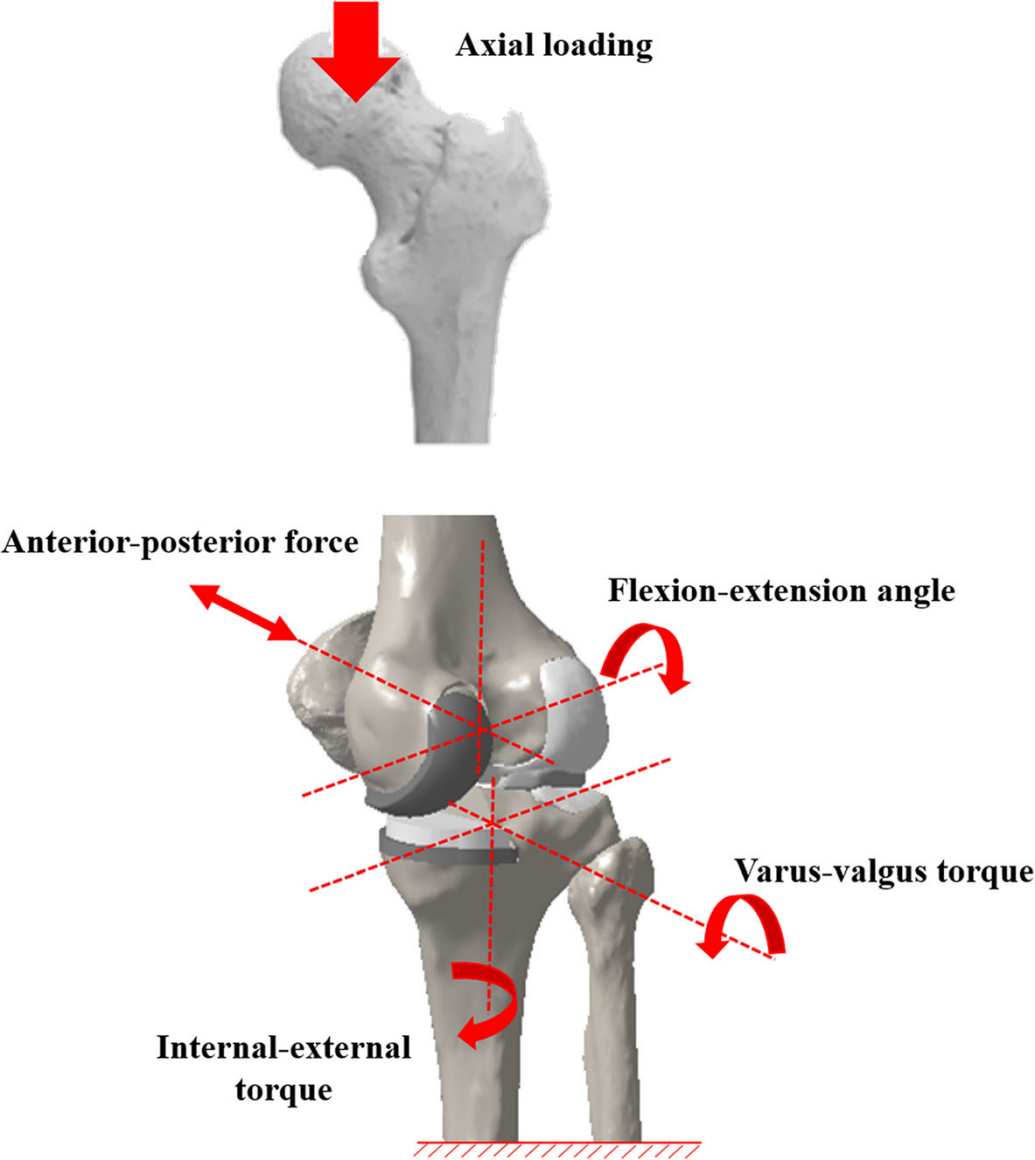
Loading condition of the UKA FE model

The femoral component and tibial insert were in contact with a coefficient of friction of 0.04 [21]. The FE simulation involved two types of loading conditions corresponding to the loads used in the model validation experiment and to predictions of loading scenarios in daily activities. An axial loading of 1150 N was applied to the model to evaluate the contact stresses and compare them with those reported in previous studies [23] (Fig. 2). The second loading condition corresponded to a deep knee bend, and squat loading was applied to evaluate the knee joint mechanics. A computational analysis was performed using an anteroposterior force applied to the femur that was based on the compressive load applied to the hip with constrained femoral internal-external (IE) rotation, free medial-lateral translation, and knee flexion, for a combination of vertical hip and quadriceps loads. Therefore, a 6-degrees-of-freedom (DOF) TF joint was developed [24, 25]. A proportional-integral-derivative controller was incorporated into the computational model to control the quadriceps in a manner similar to that in previous experiments [26]. A control system was used to calculate the instantaneous displacement of the quadricep muscles to match the target flexion profile used in the experiment. Furthermore, IE and varus-valgus torques were applied to the tibia, while the remaining tibial DOF were constrained [24, 25].

The FE models were analyzed using Abaqus version 6.11 software (Simulia, Providence, RI, USA). The contact stress and quadriceps force on the PF joint were evaluated for the FB and MB medial UKA designs.

### Statistical analyses

We performed the test at 11 time points (0.0 to 1.0 phases) for single cycles of deep-knee-bend loading conditions. To assess the two models, FB and MB, the condition of each model was compared with that of the normal knee in a pairwise manner by using non-parametric repeated-measures Friedman tests at each phase of the cycle. In this study, we used Wilcoxon rank test with Holm correction for *post hoc* comparisons to control the familywise error rate for the tests conducted within each phase of the cycle. Statistical analyses were performed using SPSS version 20.0.0 for Windows (SPSS Inc., Chicago, IL, USA). Statistical significance was set at *P* < .05.

## Results

The results of the five subject-specific FE models were compared with previous results of the same model for model validation [23]. The mean contact stresses on the medial and lateral menisci in the present and previous studies are presented in Table 4.

**Table 4:** Comparison of the average contact stresses on the menisci for the validation of the model under an axial loading condition

	Previous study [23]	Present study	Standard deviation
Medial meniscus (MPa)	2.9	3.1	0.4
Lateral meniscus (MPa)	1.4	1.5	0.6

**Fig. 3:**
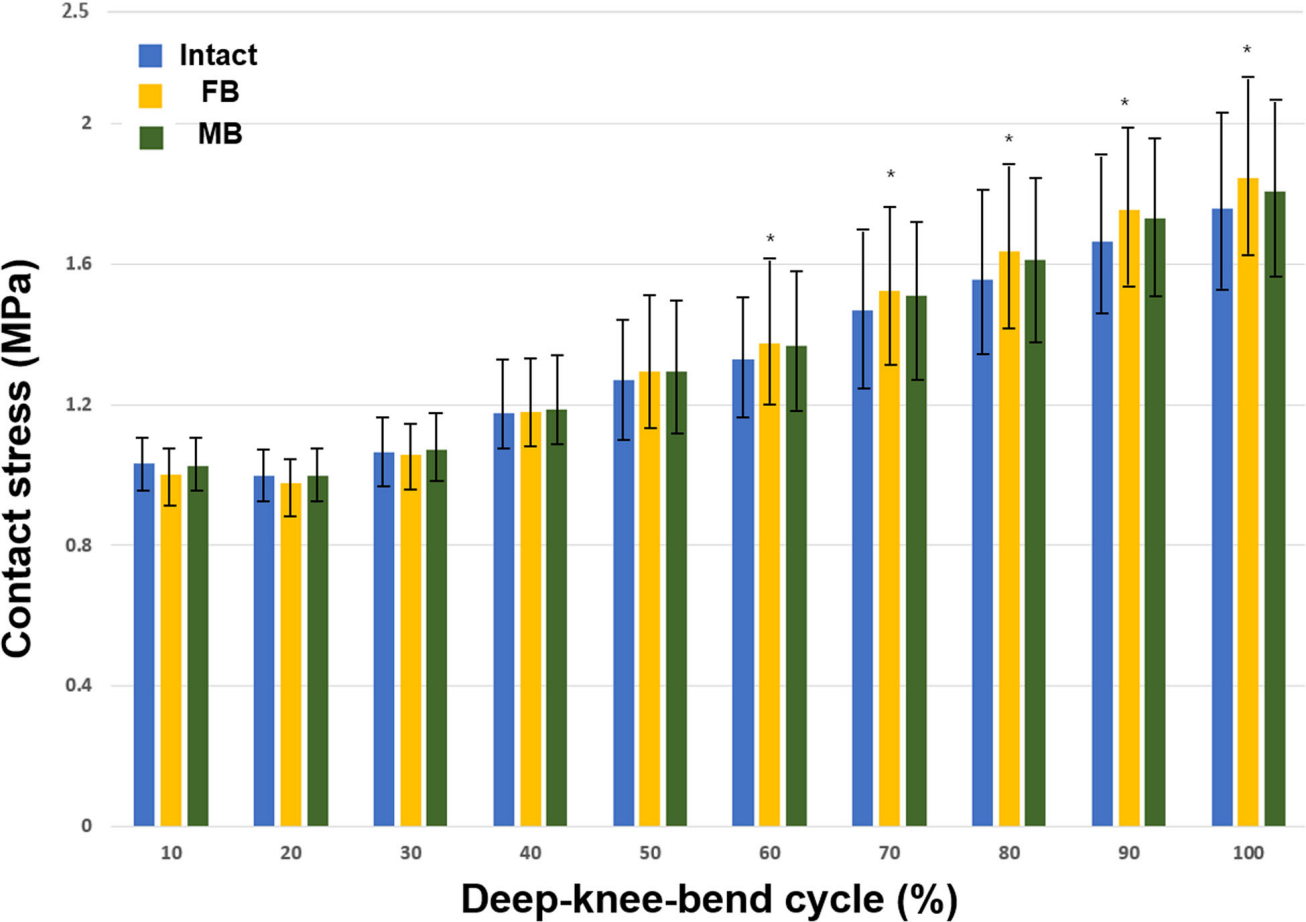
Differences in contact stress on the PF joint with intact, FB, and MB design medial UKA under the deep-knee-bend condition (*P<.05)

The minor differences may be due to the variations in geometry, such as the thicknesses of the cartilage and meniscus, between the studies. However, the consistency between the results confirms the ability of the FE model to produce reasonable results [23]. Figure 3 shows the contact stresses on the PF joint with the FB and MB medial UKA designs under the deep-knee-bend condition. No significant difference in contact stress on the PF joint was observed between the FB and MB medial UKA models and the intact model at an early flexion angle. At a larger flexion angle, the contact stress showed a significant increase of 7% (on average), which is a small but significant increase, for the FB model compared with the intact model. For the MB models, we observed an increase of 4% (on average) in contact stress. However, we found no statistically significant increment.

**Fig. 4:**
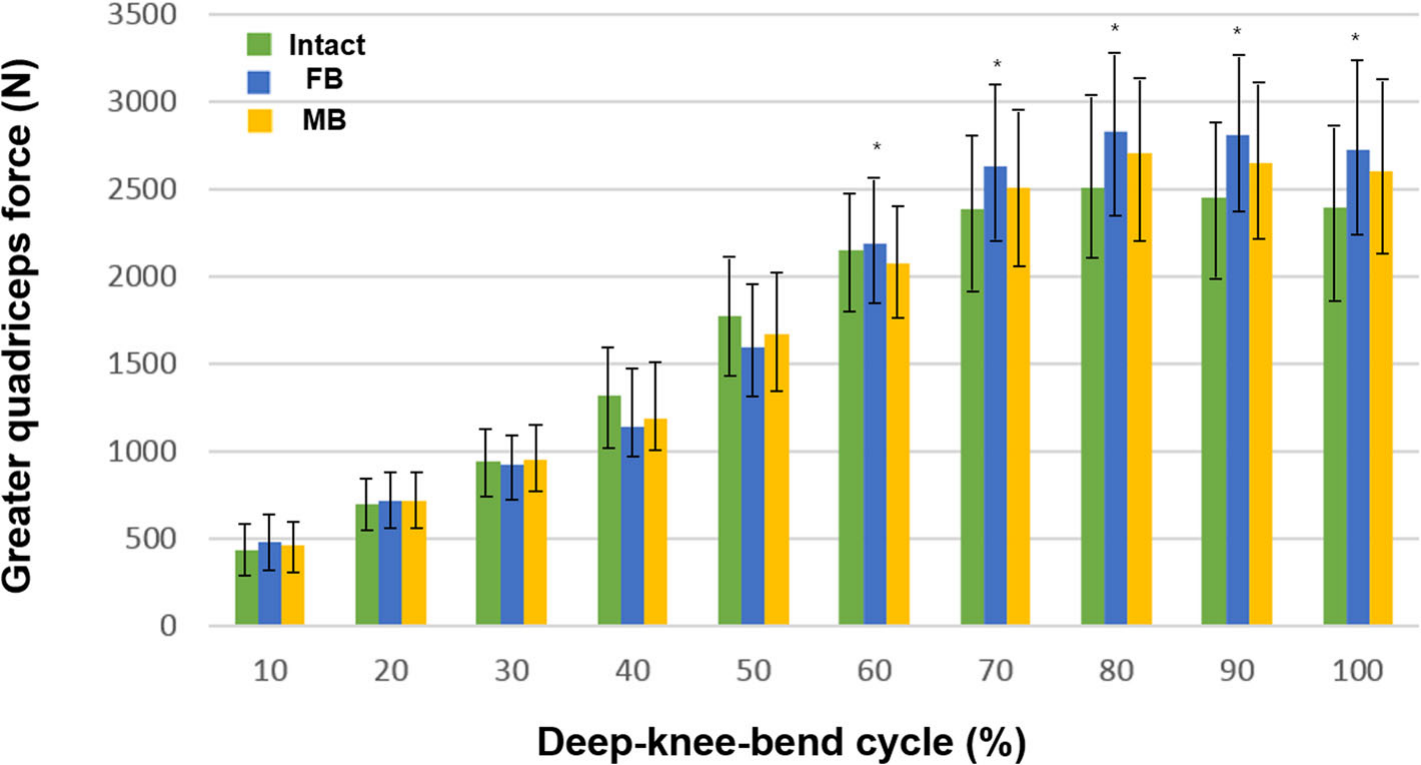
Differences in the quadriceps muscle force for intact, FB, and MB tibial insert materials under the deep-knee-bend condition (*P < .05)

The quadriceps forces exerted on the PF joint with the FB and MB medial UKA designs under the deep-knee-bend condition are shown in Fig. 4. A larger quadriceps force was needed to produce an identical flexion angle for both the FB and MB UKA designs than for the intact model. The quadriceps force rapidly increased the flexion of the knee joint in all the models. On average, the maximum quadriceps force significantly ranged from 2710 N for the MB UKA design to 2830 N for the FB UKA design. At a mid-flexion angle, the quadriceps forces were smaller for the FB and MB UKA designs than for the intact model. In addition, a lower quadriceps force was needed to produce identical flexion angles with the MB UKA design than with the FB UKA design. And at high flexion angles, a significant increase quadriceps force whit the FB model compared with the intact model. The FB and MB UKA designs required 12% and 8% (on average) more quadriceps force, respectively, than the intact model.

## Discussion

The most important finding of this study was that the contact stress on the PF joint increased less with the MB UKA design than with the FB UKA design; however, no significant difference in contact stress on the PF joint was found between the medial UKA and intact knee joints. The quadriceps force needed to produce the same flexion angle with the MB UKA design was lower than that with the FB UKA design. UKA can be performed with either an FB or MB design. In a prospective study that involved 48 patients, who were randomly assigned to either FB or MB UKA prostheses, Li et al. observed better knee kinematics and a lower incidence of radiolucency with the MB design, but the Knee Society, WOMAC, and SF-36 scores were equivalent between the two designs [27]. In another study, the range of motion, limb alignment, patient-reported outcomes, incidence of aseptic loosening, and reoperation rate were identical between the FB and MB UKA designs [28]. However, the time to reoperation and failure mode differed. Early failure due to bearing dislocation occurred with the MB design, whereas late failure due to polyethylene wear occurred with the FB design. A previous study indicated that during a ≥ 15-year follow-up period, some type of revision arthroplasty was required for 12 (15%) of 77 knees in the case of FB UKA (Miller-Galante; Zimmer) and for 10 (12%) of 79 knees in the case of MB UKA (Oxford; Biomet) [29]. No significant differences were observed in the number of knees with progressive lateral OA that required revision arthroplasty between the FB and MB UKA designs [29]. Thus, many arguments have emerged regarding the biomechanical issues of the FB and MB UKA designs. In previous studies on the progression of OA after UKA, the radiological assessment was neither blinded nor randomized [27].

The advantage of FE analysis is that the impact of the UKA design can be determined without external variables [30]. Most in vitro biomechanical studies have involved evaluations using aged cadaveric subjects with loosening between the specimen and the device, and tissue attenuation, which can occur owing to the successive loading in mechanical testing [26]. An intact joint model was the foundation of this study and involved FEM validation steps. The results exhibited good agreement with those of previous computational studies [23, 31]. Therefore, the UKA models used in the present study and related analyses are considered reliable.

Kozinn and Scott proposed that UKA should not be offered to patients with PF joint arthritis for optimal results [32]. This sparked a contentious debate on PF joint disease because other authors demonstrated only a weak correlation between PF degenerative changes and anterior knee pain [11, 33].

In addition, owing to the differences in the design and biomechanics of the FB UKA model, damage to the PF joint has traditionally been a contraindication. Lim et al. recently showed that the presence of significant preoperative radiological PF disease does not affect long-term implant success, and patients had excellent postoperative functional outcomes for 10 years [34]. In the present study, the MB UKA design produced a smaller increase in contact stress on the PF joint than the intact joint and FB UKA design models. The results of previous studies on MB UKA indicated that the presence of PF degeneration does not compromise clinical outcomes because the implant is believed to be more patella friendly owing to better kinematics, which supports our results [34, 35]. Moreover, although the contact stress on the PF joint increased with both the FB and MB UKA designs, the increase was not statistically significant. Biomechanical studies have indicated that the progression of arthritis of the PF joint typically does not necessitate revision.

The quadriceps force needed to produce a squatting motion was greater for the FB design than for the MB design (by as much as 120 N for knee flexion angles > 100°). Thus, increased quadriceps strength leads to improved functional performance [36]. As patients who have undergone OA and knee arthroplasty experience significant quadriceps weakness, the FB UKA design, which increases the required quadriceps force, can result in more difficulty for patients to walk, kneel, or perform a deep knee bend [37]. This agrees with the results of a previous in vitro study in which a UKA model required less quadriceps force at a mid-flexion angle than an intact model [27].

From a biomechanical viewpoint, our results indicate that the risk of progressive OA of the PF joint can be reduced with the MB UKA design because it preserves the normal biomechanical effect, in contrast to the FB UKA design. In addition, the MB UKA design requires a lower quadriceps force and makes it easier for recipients to kneel, squat, or rise from a chair.

The three strengths of our study should be highlighted. First, a well-validated setup that accounted for numerous previous results was used. Second, in contrast to previous UKA studies, the present study included the tibia, femur, and related soft tissues in the FE model. Third, in contrast to the current biomechanical UKA models, the model used in this study included the deep-knee-bend and squat loading conditions, rather than the simple vertical static loading condition.

Despite these strengths, this study had certain limitations. First, the results did not predict clinical results or patient satisfaction. Second, the computational model was developed using data from four male subjects and one female subject. Using data from subjects of various ages would improve the validity of the results, as it would increase the diversity of the knee joint geometry. However, in this study, our objective was to evaluate the biomechanical effect of UKA in young individuals. Third, the bony structures were assumed to be rigid. In reality, the bone is composed of cortical and cancellous tissues. However, the main purpose of the study was not to evaluate the effects of different prostheses on bone. In addition, this assumption had a minimal influence on the results of the study because the bone stiffness exceeds that of the relevant soft tissues [23]. Finally, the simulation only involved a deep knee bend; thus, simulations involving rising from or sitting on chairs, climbing/descending stairs, and squatting should be performed in future investigations.

In conclusion, this study provides biomechanical evidence that degenerative changes in the PF joint should not be considered an absolute contraindication to treatment with medial UKA. In addition, UKA is not problematic even in PF joints with OA, unless accompanied by anterior knee pain, because no significant difference in contact stress was observed.

## Data Availability

Not applicable.

## Abbreviations

UKA: Unicompartmental knee arthroplasty
OA: Osteoarthritis
PF: Patellofemoral
FB: Fixed-bearing
MB: Mobile-bearing
TF: Tibiofemoral
IE: Internal-external
DOF: Degree of freedom
